# Clinical characteristics and risk factors of polymicrobial *Staphylococcus aureus* bloodstream infections

**DOI:** 10.1186/s13756-020-00741-6

**Published:** 2020-05-27

**Authors:** Cheng Zheng, Shufang Zhang, Qingqing Chen, Li Zhong, Tiancha Huang, Xijiang Zhang, Kai Zhang, Hongwei Zhou, Jiachang Cai, Linlin Du, Changming Wang, Wei Cui, Gensheng Zhang

**Affiliations:** 1 Department of Critical Care Medicine, Second Affiliated Hospital, Zhejiang University School of Medicine, Hangzhou, 310009 China; 2 Department of Critical Care Medicine, Taizhou Municipal Hospital, Taizhou, 318000 Zhejiang China; 3 Department of Cardiology, Second Affiliated Hospital, Zhejiang University School of Medicine, Hangzhou, 310009 China; 4 Department of Critical Care Medicine, Taizhou Enze Medical Center (Group) Enze Hospital, Taizhou, 318050 Zhejiang China; 5 Department of Critical Care Medicine, Huzhou First People’s Hospital, Huzhou, 313000 Zhejiang China; 6 Clinical Microbiology Laboratory, Second Affiliated Hospital, Zhejiang University School of Medicine, Hangzhou, 310009 China

**Keywords:** *Staphylococcus aureus*, Bacteremia, Polymicrobial *Staphylococcus aureus* bloodstream infections, Risk factors, Mortality

## Abstract

**Background:**

Although *Staphylococcus aureus* bloodstream infections (SA-BSI) are a common and important infection, polymicrobial SA-BSI are infrequently reported. The aim of this study was to investigate the clinical characteristics and risk factors of polymicrobial SA-BSI in comparison with monomicrobial SA-BSI.

**Methods:**

A single-center retrospective observational study was performed between Jan 1, 2013, and Dec 31, 2018 at a tertiary hospital. All patients with SA-BSI were enrolled, and their clinical data were gathered by reviewing electronic medical records.

**Results:**

A total of 349 patients with SA-BSI were enrolled including 54 cases (15.5%) with polymicrobial SA-BSI. In multivariable analysis, burn injury (adjusted odds ratio [OR], 7.04; 95% confidence interval [CI], 1.71–28.94), need of blood transfusion (aOR, 2.72; 95% CI, 1.14–6.50), use of mechanical ventilation (aOR, 3.11; 95% CI, 1.16–8.30), the length of prior hospital stay (aOR, 1.02; 95% CI, 1.00–1.03), and pneumonia as primary site of infection (aOR, 4.22; 95% CI, 1.69–10.51) were independent factors of polymicrobial SA-BSI. In comparison with monomicrobial SA-BSI, patients with polymicrobial SA-BSI had longer length of ICU stay [median days, 23(6.25,49.25) vs. 0(0,12), *p* < 0.01] and hospital stay [median days, 50(21.75,85.75) vs. 28(15,49), *p* < 0.01], and showed a higher 28-day mortality (29.6% vs. 15.3%, *p* = 0.01).

**Conclusions:**

Burn injury, blood transfusion, mechanical ventilation, the length of prior hospital stay, and pneumonia as a primary site of infection are independent risk factors for polymicrobial SA-BSI. In addition, patients with polymicrobial SA-BSI might have worse outcomes compared with monomicrobial SA-BSI.

## background

Due to their potentially serious consequences, bloodstream infections (BSI) are a growing worldwide concern [1]. BSI can be caused by a wide variety of microorganisms, and the most common organisms were Coagulase-negative *staphylococci* (CNS), *Staphylococcus aureus* (*S. aureus*), *Enterococci*, and *Candida* species [2]. *S. aureus* is the second most common cause of BSI, which also serves as the most important cause of BSI-associated death [3, 4]. Most of BSI are monomicrobial, but the trend of polymicrobial BSI is rising with a range of 6% ~ 34% among BSI [2, 5, 6]. Polymicrobial BSI is generally associated with a higher acute physiology and chronic health evaluation (APACHE) II scores, prolonged ICU and hospital stay, and a more severe prognosis than monomicrobial BSI in adults [5–9]. In these previous studies [5–11], some limitations also existed as follows: (1) The clinical significance and outcomes of polymicrobial versus monomicrobial BSI were indeed investigated, but few reports focused on a specific pathogen. Thus, the specific clinical features and outcomes between polymicrobial SA-BSI and monomicrobial SA-BSI are still largely unknown. (2) In a previous study [10], patients with polymicrobial SA-BSI often had a biliary source and had a worse prognosis, and independent risk factors for polymicrobial SA-BSI included neutropenia, biliary tract catheters, and intra-abdominal infection. A bias was also pointed out that a high proportion of biliary tract diseases (7%) was observed in their institution [10]. (3) Another study illustrated that SA-BSI was usually monomicrobial, and soft tissue was the most common source [11]. Unfortunately, this study did not investigate clinical characteristics and the risk factors for polymicrobial SA-BSI. (4) However, these two studies [10, 11] focused on Korean and American population respectively, and there were no studies focused on Chinese population at present. Thus, the clinical outcomes between polymicrobial SA-BSI and monomicrobial SA-BSI are still controversial. Herein, we conducted the retrospective study on polymicrobial SA-BSI to provide more information of the clinical characteristics and risk factors of polymicrobial SA-BSI.

## Materials and methods

### Patients and study design

This single-center retrospective cohort study was conducted from January 2013 to December 2018 in the Second Affiliated Hospital, Zhejiang University School of Medicine, a 3200-bed tertiary-level healthcare facility in Hangzhou, China. The present study received human research ethics approval (No. 2019–194) from the Ethics Committee of the Second Affiliated Hospital, Zhejiang University School of Medicine. Due to the retrospective nature of the study, the Ethics Committee determined that no patient consent was required. In addition, a statement of permission from patients for submission was not required as the study did not include any personal information.

**Fig. 1:**
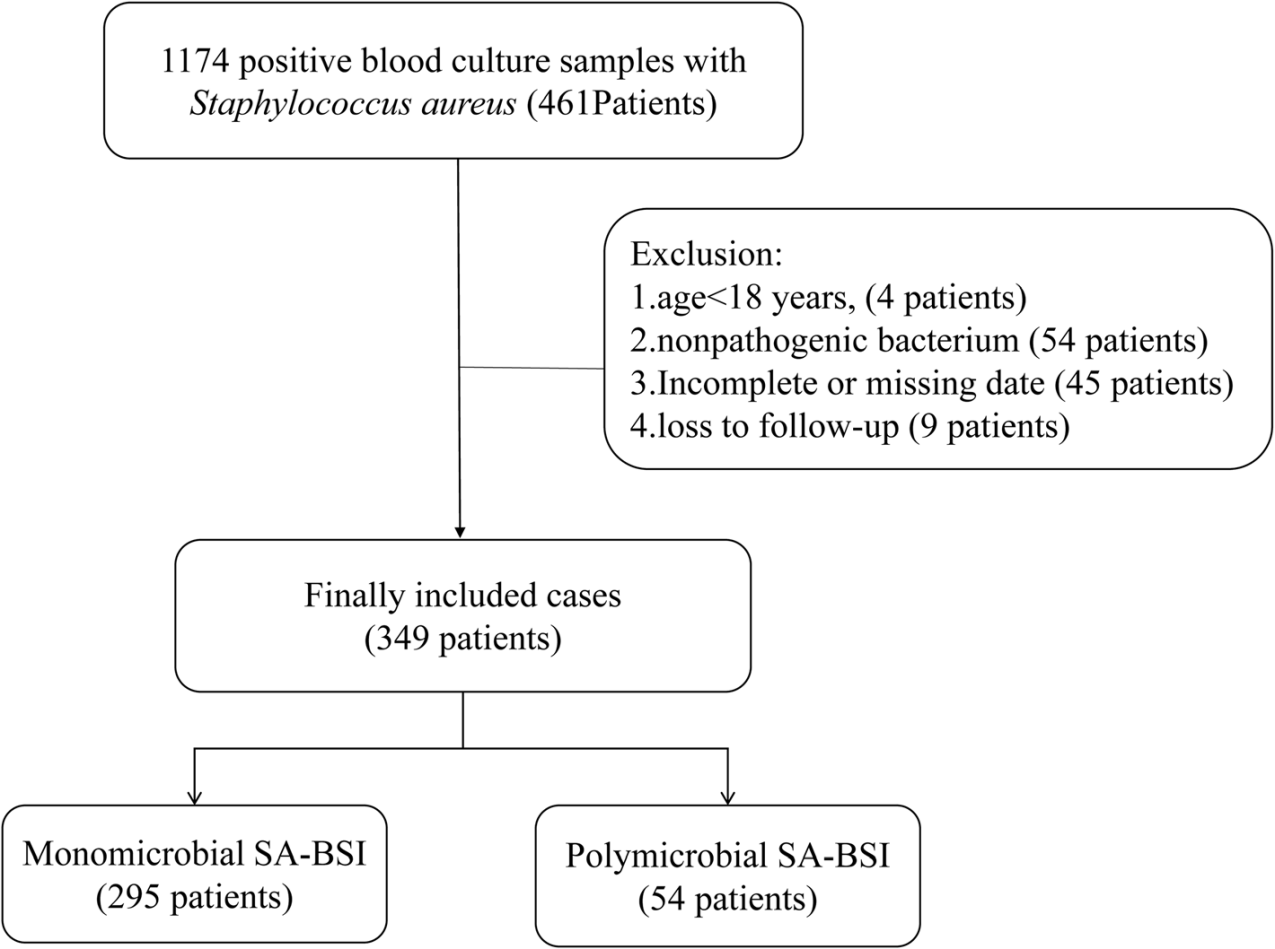
Flowchart of study participant enrollment. Abbreviations: SA-BSI, *Staphylococcus aureus* bloodstream infections

If any microorganisms besides *S. aureus* were found in the same blood culture, the cases were retained. If only *S. aureus* was found in multiple blood cultures of the same patient, the patients were included only one time when they happened with SA-BSI at the first time. Exclusion criteria were as follows: a) Age < 18 years old; b) *S. aureus* considered as nonpathogenic bacterium (In bilateral double bottles blood culture, *S. aureus* was only cultured in one bottle, and the culture time was more than 48 h); c) Cases data were incomplete or missing; d) Loss to follow-up. Common skin contaminant organisms (eg, *Bacillus* spp*.*, *Corynebacterium* spp*.*, *Micrococcus* spp*.*, *Streptococci*, *Lactobacillus* spp. and CNS) were considered pathogens only when they were present in two or more consecutive blood cultures from separate blood draws. Thus, a total of 1174 blood culture specimens containing *S. aureus* were initially included, and 349 cases were finally recruited with 54 cases of polymicrobial SA-BSI and 295 cases of monomicrobial SA-BSI (Fig. 1).

### Data collection

The patients’ data were collected by reviewing electronic medical records. We recorded demographic data including age and gender, the clinical data including underlying diseases, Sequential Organ Failure Assessment (SOFA) score, Pitt bacteremia score, Charlson Comorbidity Index (CCI) score, APACHE II score in the first 24 h following the onset of BSI, the hospitalization wards, nosocomial infection or not, previous exposures (prior hospital stay, previous treatment such as surgical procedures, immunosuppressive agents, chemotherapeutic agents, radiation therapy, parenteral nutrition, mechanical ventilation, renal replacement therapy, blood transfusion), and outcomes (length of hospital stay, length of ICU stay, occurrence of septic shock and 28-day mortality). The microbiological data were also collected including likely source of BSI (identified by treating doctors), mono-microorganism/poly-microorganisms, and sensitivity to antibiotics. If the source of a BSI could not be attributed to any known source, it was classified as a primary BSI [12].

### Species identification and antibiotic sensitivity test

Blood was cultured using a BacT/ALERT 3D system (Becton-Dickinson, Sparks, MD, USA) in the microbiology laboratory. Species identification was performed using Bruker Daltonics DataAnalysis. Antibiotic susceptibility testing was performed using the VITEK 2 (Card number: AST-GN16; AST-GP67) system or the Kirby-Bauer Disk Diffusion method (Oxoid, UK) according to the recommendations proposed by the Clinical and Laboratory Standards Institute (CLSI).

### Definitions

Diagnosis of SA-BSI was based on CDC definitions for Bloodstream Infection Events [12]. Onset of BSI was defined as the time when the blood culture was collected. Polymicrobial SA-BSI was defined as the simultaneous isolations of *S. aureus* and one or more other organisms from blood cultures [10]. Nosocomial BSI was defined as the first positive blood culture obtained ≥48 h after hospital admission and with no evidence of infection at admission [13, 14]. Infective endocarditis was defined using the modified Duke criteria [15]. Appropriate antimicrobial therapy was considered early when administered within 24 h after the first blood culture that yielded *S. aureus* had been obtained [16], whereas therapy was considered delayed when more than 24 h had elapsed [16]. Neutropenia was defined as absolute neutrophil counts of 1000/mm^3^ or below when bacteremia occurred. Sepsis and septic shock were defined according to the new definition of Sepsis-3 [17]. Secondary BSI was defined as a BSI that is thought to be seeded from a site-specific infection at another body site [12].

### Statistical analysis

Statistical analysis was performed with SPSS 20.0 software (IBM Corp, Armonk, NY, USA). Continuous variables were presented as mean ± standard deviation if normally distributed, and as median and interquartile range (IQR) if non-normally distributed. Continuous variables were compared by Student t test or Mann-Whitney U test and enumeration variables were compared by Pearson χ^2^ or Fisher exact test, where appropriate. Variables that had significance at a *p* < 0.05 level in the univariate analysis were considered candidates for the building of stepwise logistic regression multivariable models. A two-tailed *p* < 0.05 was considered statistically significant.

## Results

### Demographic and clinical characteristics

The demographic and clinical characteristics of these patients were summarized in Table 1. The median age was 59 years (IQR, 45.5–68), and 69.6% (243/349) were male. Trauma was the most common comorbidity (20.9%), followed by diabetes mellitus (20.1%). There were no significant differences in age or gender between the two groups. In terms of co-morbidities, a significantly high percentage of trauma, burn injuries, or cerebrovascular accident was observed in patients with polymicrobial SA-BSI (all *p* < 0.05). In comparison with monomicrobial SA-BSI, patients with polymicrobial SA-BSI presented a more severe condition, evidenced by a higher APACHE II score (median, 15 vs. 12, *p* < 0.01), a higher SOFA score (median, 5 vs. 3, *p* = 0.01) and a higher Pitt Bacteremia Score (median, 3.5 vs 1, *p* < 0.01), and displayed more need of ICU admission (70.4% vs. 31.5%, *p* < 0.01) or invasive mechanical ventilation (66.7% vs. 25.8%, *p* < 0.01). Compared with monomicrobial SA-BSI, patients with polymicrobial SA-BSI had a greater proportion of receiving parenteral nutrition (57.4% vs. 30.8%, *p* < 0.01), more need of blood transfusion (38.9% vs. 12.5%, *p* < 0.01), significant increases in central line indwelling and urinary catheter indwelling (64.8% vs. 44.7%, *p* < 0.01; and 79.6% vs. 44.7%, *p* < 0.01), more surgery (59.3% vs 31.2%, *p* < 0.01), and more nosocomial infections (96.3% vs. 72.5%, *p* < 0.01). In addition, a longer hospital stay before BSI onset was observed in patients with polymicrobial SA-BSI than monomicrobial SA-BSI (median days, 13 vs. 7, *p* < 0.01).

**Table 1:** Baseline characteristics of patients with polymicrobial and monomicrobial *Staphylococcus aureus* bloodstream infection

Characteristics	Total (*n* = 349)	Mono-SA-BSI (*n* = 295)	Poly-SA-BSI (*n* = 54)	*p*-value
Age, median years (IQR)	59.00 (45.50,68.00)	60.00 (48,68.00)	51.00 (40.50,69.00)	0.06
Male sex	243 (69.6%)	202 (68.5%)	41 (75.9%)	0.27
Co-morbidities				
Diabetes mellitus	70 (20.1%)	61 (20.7%)	9 (16.7%)	0.50
Chronic kidney disease	31 (8.9%)	27 (9.2%)	4 (7.4%)	0.88
Chronic liver disease	16 (4.6%)	14 (4.7%)	2 (3.7%)	1
COPD or Severe asthma	7 (2%)	7 (2.4%)	0 (0%)	0.60
Chronic cardiac insufficiency	25 (7.2%)	22 (7.5%)	3 (5.6%)	0.83
Solid tumor	57 (16.3%)	48 (16.3%)	9 (16.7%)	0.94
Trauma	73 (20.9%)	54 (18.3%)	19 (35.2%)	*0.01*
Burn injury	45 (12.9%)	30 (10.2%)	15 (27.8%)	*0.00*
Cerebrovascular accident	39 (11.2%)	28 (9.5%)	11 (20.4%)	*0.02*
CCI, median (IQR)	3 (1,5)	3 (2,5)	2 (1,4.25)	0.08
APACHE II score, median (IQR)	12 (9,17)	12 (8,16)	15 (11,22)	*0.00*
SOFA score, median (IQR)	4 (2,6)	3 (2,5)	5 (2.75,10)	*0.01*
Pitt Bacteremia Score, median (IQR)	2 (1,4)	1 (1,3)	3.5 (2,6)	*0.00*
Hospitalization ward				
ICU stay	131 (37.5%)	93 (31.5%)	38 (70.4%)	*0.00*
Previous treatment				
Parenteral nutrition	122 (35%)	91 (30.8%)	31 (57.4%)	*0.00*
Mechanical ventilation	112 (32.1%)	76 (25.8%)	36 (66.7%)	*0.00*
Antibiotic exposure	302 (86.5%)	251 (85.1%)	51 (94.4%)	0.06
Surgery	124 (35.5%)	92 (31.2%)	32 (59.3%)	*0.00*
Chemotherapy/radiation	13 (3.7%)	13 (4.4%)	0 (0%)	0.23
Renal replacement therapy	43 (12.3%)	37 (12.5%)	6 (11.1%)	0.77
Blood transfusion	58 (16.6%)	37 (12.5%)	21 (38.9%)	*0.00*
Invasive devices				
central line catheter	167 (47.9%)	132 (44.7%)	35 (64.8%)	*0.01*
Indwelling urinary catheter	175 (50.1%)	132 (44.7%)	43 (79.6%)	*0.00*
Intraperitoneal drainage	33 (9.5%)	26 (8.8%)	7 (13%)	0.34
Prior hospital stay, median days (IQR)	8 (2,20)	7 (1,19)	13 (7,32.5)	*0.00*
Nosocomial infection	266 (76.2%)	214 (72.5%)	52 (96.3%)	*0.00*
Neutropenia	7 (2.0%)	6 (2.0%)	1 (1.9%)	1

*Abbreviations*: *IQR* Interquartile range, *COPD* Chronic obstructive pulmonary disorder, *CCI* Charlson Comorbidity Index, *SOFA* Sequential organ failure assessment, *APACHE* Acute physiology and chronic health evaluation, *ICU* Intensive care unit, *SA-BSI Staphylococcus aureus* bloodstream infection

### Biological indicators

A comparison of biological indicators between polymicrobial SA-BSI and monomicrobial SA-BSI was shown in Table 2. In comparison with monomicrobial SA-BSI, patients with polymicrobial SA-BSI had a lower hematocrit (median %, 26.95 vs. 29.2, *p* < 0.01), a worse liver function evidenced by significant increases in Glutamic-pyruvic transaminase (GPT) (median U/L, 41 vs. 30, *p* = 0.01), Glutamic-oxaloacetic transaminase (GOT) (median U/L, 36 vs. 28, *p* < 0.01) and Lactic dehydrogenase (LDH) (median U/L, 343 vs. 248, *p* < 0.01). However, there was no significant difference in procalcitonin between the two groups.

**Table 2:** Comparison of biological indicators between groups of monomicrobial SA-BSI and polymicrobial SA-BSI

Biological indicators	Total (*n* = 349)	Mono-SA-BSI (*n* = 295)	Poly-SA-BSI (*n* = 54)	*p*-value
Temperature (°C) (IQR)	39.0 (38.6,39.5)	39 (38.6,39.5)	39 (38.575,39.5)	0.51
Blood routine test				
WBC(× 10^9^/L) (IQR)	10.1 (6.8,14.35)	10.3 (6.9,14.2)	9.75 (5.5,16.65)	0.94
Hematocrit (%) (IQR)	28.9 (24.05,33.5)	29.2 (24.6,33.9)	26.95 (22.6,31.2)	*0.01*
Platelet (×10^9^/L) (IQR)	171 (102.5247.5)	175 (101,248)	145.5 (103,248)	0.37
ANC (IQR)	8.15 (5.56,12.74)	8.1 (5.6,12.35)	10.545 (5.44,15.79)	0.13
Liver and kidney function				
Albumin (g/L) (mean ± S.D.)	29.56 ± 5.66	30.216 ± 5.48	29.44 ± 5.69	0.36
GPT (U/L) (IQR)	31 (17,57)	30 (17,53)	41 (24.5,73)	*0.01*
GOT (U/L) (IQR)	30 (21,50)	28 (20,47)	36 (27.5,60.25)	*0.00*
ALP (U/L) (IQR)	101 (74,151)	103 (74,150)	99.5 (76.75,152.25)	0.97
γ-GT (U/L) (IQR)	45 (26.0,95)	44 (25.0,96.0)	56.0 (27.25,89)	0.42
LDH (U/L) (IQR)	254 (197,342)	248 (195,321)	343 (227.5405)	*0.00*
TBil (μmol/L) (IQR)	12.8 (9,20.35)	12.7 (9,19.6)	14.85 (9.38,26.43)	0.26
SCr (μmol/L) (IQR)	59 (44.5,95.5)	59 (45,96)	65.5 (42.25,96.25)	0.94
PCT (ng/ml) (IQR)	0.46 (0.18,1.31)	0.46 (0.18,1.29)	0.53 (0.22,2.08)	*0.36*

*Abbreviations*: *SA-BSI Staphylococcus aureus* bloodstream infections, *IQR* Interquartile range, *WBC* White blood count, *ANC* Absolute neutrophil count, *GPT* Glutamic-pyruvic transaminase, *GOT* Glutamic-oxaloacetic transaminase, *ALP* Alkaline phosphatase, *γ-GT* Gamma glutamyl transpeptidase, *LDH* Lactic dehydrogenase, *TBil* Total bilirubin, *SCr* Serum creatinine, *PCT* Procalcitonin

### Independent risk factors for polymicrobial SA-BSI

As shown in Table 3, multivariate logistic regression model analysis showed that the independent risk factors of polymicrobial SA-BSI were burn injury (adjusted odds ratio [aOR], 7.04; 95% confidence interval [CI], 1.71–28.94), prior blood transfusion (aOR, 2.72; 95% CI, 1.14–6.50), mechanical ventilation (aOR, 3.11; 95% CI, 1.16–8.30), pneumonia as a primary site of infection (aOR, 4.22; 95% CI, 1.69–10.51), and the days of prior hospital stay before onset of BSI (aOR, 1.02; 95% CI, 1.00–1.03).

**Table 3:** Multivariable logistic regression of factors associated with polymicrobial *Staphylococcus aureus* bloodstream infections

Variable	Unadjusted OR(95%CI)	*p*-value	Adjusted OR(95%CI)	*p*-value
Co-morbidities				
Trauma	2.42 (1.29,4.56)	0.01		
Burn injury	3.40 (1.68,6.88)	0.00	7.04 (1.71,28.94)	*0.01*
Cerebrovascular accident	2.44 (1.13,5.26)	0.02		
APACHE II score	1.08 (1.04,1.12)	0.00		
SOFA score	1.11 (1.04,1.18)	0.00		
Pitt Bacteremia Score	1.29 (1.15,1.44)	0.00		
ICU stay	5.16 (2.74,9.72)	0.00		
Previous treatment				
Parenteral nutrition	3.02 (1.67,5.47)	0.00		
Previous surgery	3.21 (1.77,5.83)	0.00		
Prior Blood transfusion	4.44 (2.32,8.47)	0.00	2.72 (1.14,6.50)	*0.03*
Central venous catheter	2.28 (1.24,4.16)	0.01		
Mechanical ventilation	5.76 (3.09,10.75)	0.00	3.11 (1.16,8.30)	*0.02*
Indwelling urinary catheter	4.83 (2.40,9.73)	0.00		
Nosocomial infection	9.84 (2.34,41.34)	0.00		
Prior hospital stay	1.02 (1.01,1.03)	0.01	1.02 (1.00,1.03)	*0.02*
Source of BSIs				
Pneumonia	2.62 (1.44,4.78)	0.00	4.22 (1.69,10.51)	*0.00*
Central venous catheter	0.31 (0.11,0.88)	0.03		
MRSA	2.36 (1.14,4.88)	0.02		

*Abbreviations*: *CCI* Charlson Comorbidity Index, *SOFA* Sequential organ failure assessment, *APACHE* Acute physiology and chronic health evaluation, *ICU* Intensive care unit, *BSIs* Bloodstream infections, *MRSA* Methicillin-resistant *Staphylococcus aureus*, *OR* Odds ratio, *CI* Confidence interval

### Bacteriology and sources of polymicrobial SA-BSI

**Fig. 2:**
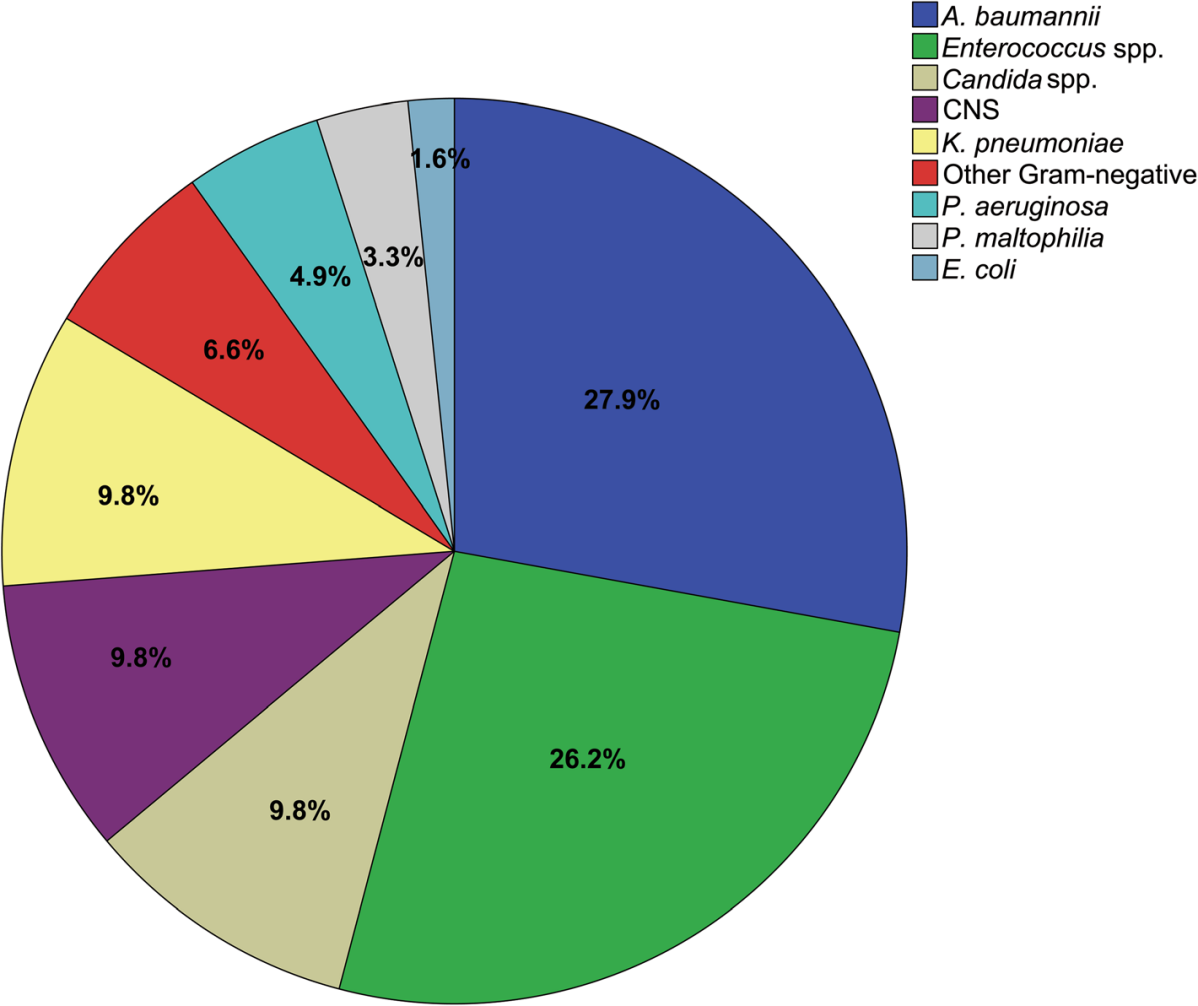
Distribution of the additional organisms in polymicrobial *Staphylococcus aureus* bloodstream infections. Abbreviations: *E. coli*, *Escherichia coli*; *A. baumannii, Acinetobacter baumannii*; CNS, Coagulase-negative *Staphylococcus*; *K. pneumoniae, Klebsiella pneumoniae*; *P. aeruginosa*, *Pseudomonas aeruginosa*; *P. maltophilia*, *Pseudomonas maltophilia*

The isolated pathogens were shown in Fig. 2. A total of 61 microorganisms other than *S. aureus* were isolated from 54 polymicrobial SA-BSI cases, with two microorganisms accounting for 87% (47/54) and three microorganisms for 13% (7/54). The most common co-pathogen was Gram-negative bacteria (54.1%), followed by Gram-positive bacteria (36.1%) and fungi (9.8%). In terms of a specific microorganism, the most frequent pathogen was *Acinetobacter baumannii* (*A. baumannii*) (27.9%), followed by *Enterococcus* spp. (26.2%). *Candida* spp. was observed in only 11.1% (6/54) of patients, representing 9.8% of all isolates (Fig. 2).

The source of SA-BSI was mainly from pneumonia (26.6%, 93/349), followed by skin/soft tissue infection (24.6%, 86/349), and central venous catheter (18.6%, 65/349) (Table 4). Compared with monomicrobial SA-BSI, polymicrobial SA-BSI had more source from pneumonia (44.4% vs. 23.4%, *p* < 0.01), in which the polymicrobial SA-BSI caused by hospital-acquired pneumonia was significantly higher than that caused by community-acquired pneumonia (91.7% vs. 8.3%, *p* = 0.02), whereas monomicrobial SA-BSI had more source from central venous catheter (20.7% vs. 7.4%, *p* = 0.04).

**Table 4:** Comparison of the microbiological characteristics with monomicrobial SA-BSI and polymicrobial SA-BSI

	Total (*n* = 349)	Mono-SA-BSI (*n* = 295)	Poly-SA-BSI (*n* = 54)	*p*-value
Source of BSIs				
Pneumonia	93 (26.6%)	69 (23.4%)	24 (44.4%)	*0.00*
Hospital-acquired pneumonia	69 (74.2%)	47 (68.1%)	22 (91.7%)	*0.02*
Community-acquired pneumonia	24 (25.8%)	22 (31.9%)	2 (8.3%)	
Skin and Soft tissue infection	86 (24.6%)	69 (23.4%)	16 (29.6%)	0.33
Central venous catheter	65 (18.6%)	61 (20.7%)	4 (7.4%)	*0.04*
Intra-abdominal	42 (12%)	34 (11.5%)	8 (14.8%)	0.50
Primary BSI	28 (7.7%)	27 (9.2%)	1 (1.9%)	0.10
Bone and joint	14 (4.0%)	14 (4.7%)	0 (0%)	0.14
Endocarditis	11 (3.2%)	11 (3.7%)	0 (0%)	0.23
Urinary tract infection	9 (2.6%)	8 (2.7%)	1 (1.9%)	0.58
Others*	2 (0.6%)	2 (0.7%)	0 (0%)	0.71
MRSA	236 (67.6%)	192 (65.1%)	44 (81.5%)	*0.02*
Delayed antibiotic therapy	29 (8.3%)	24 (8.1%)	5 (9.3%)	0.78
Antibiotic resistance^a^				
Cefoxitin (115 vs 25)^b^	114 (81.4%)	89 (77.4%)	25 (100%)	*0.01*
Ciprofloxacin (237 vs 42)^b^	132 (47.3%)	96 (40.5%)	36 (85.5%)	*0.00*
Clindamycin (295 vs 54)^b^	127 (36.4%)	110 (37.3%)	17 (31.5%)	0.42
Erythromycin (295 vs 54)^b^	203 (58.2%)	167 (56.6%)	36 (66.7%)	0.17
Gentamicin (295 vs 54)^b^	58 (16.6%)	50 (16.9%)	8 (14.8%)	0.71
Levofloxacin (261 vs 49)^b^	161 (51.9%)	118 (45.2%)	43 (87.8%)	*0.00*
Linezolid (293 vs 54)^b^	46 (13.3%)	37 (12.6%)	9 (16.7%)	0.42
Moxifloxacin (287 vs 51)^b^	158 (46.7%)	117 (40.8%)	41 (80.4%)	*0.00*
Nitrofurantoin (255 vs 50)^b^	1 (0.3%)	1 (0.4%)	0 (0%)	1
Oxacillin (295 vs 54)^b^	236 (67.6%)	192 (65.1%)	44 (81.5%)	*0.02*
Penicillin (295 vs 54)^b^	333 (95.4%)	279 (94.6%)	54 (100%)	0.08
Quinupristin/Dalfopristin (295 vs 54)^b^	44 (12.6%)	36 (12.2%)	8 (14.8%)	0.60
Rifampin (242 vs 43)^b^	9 (3.2%)	8 (3.3%)	1 (2.3%)	1
Tetracycline (230 vs 50)^b^	148 (52.9%)	112 (48.7%)	36 (72%)	*0.00*
Vancomycin (278 vs 49)^b^	48 (14.3%)	39 (13.6%)	9 (18.4%)	0.38

*oromaxillo-facial region and prosthetic device

^a^Not all agents listed tested in all isolates

^b^The numbers in parentheses represent the total numbers of *Staphylococcus aureus* isolates performed susceptibility test

*Abbreviations*: *BSIs* Bloodstream infections, *MRSA* Methicillin-resistant *Staphylococcus aureus*, *SA-BSI Staphylococcus aureus* bloodstream infections

### Antibiotic resistance and appropriate therapy

In comparison with monomicrobial SA-BSI, the ratio of resistance of *Staphylococcus aureus* to cefoxitin, ciprofloxacin, levofloxacin, moxifloxacin, oxacillin, and tetracycline were significantly higher in polymicrobial SA-BSI groups (Table 4). Of note, methicillin-resistant *Staphylococcus aureus* (MRSA) was significantly more frequent in patients with polymicrobial than monomicrobial SA-BSI (81.5% vs. 65.1%, *p* = 0.02). In addition, a total of 8.3% (29/349) patients did not receive appropriate therapy within 24 h after the release of antibiotic susceptibility results, but there was no difference between the two groups (8.1% vs. 9.3%, *p* = 0.78) (Table 4).

### Outcomes

**Fig. 3:**
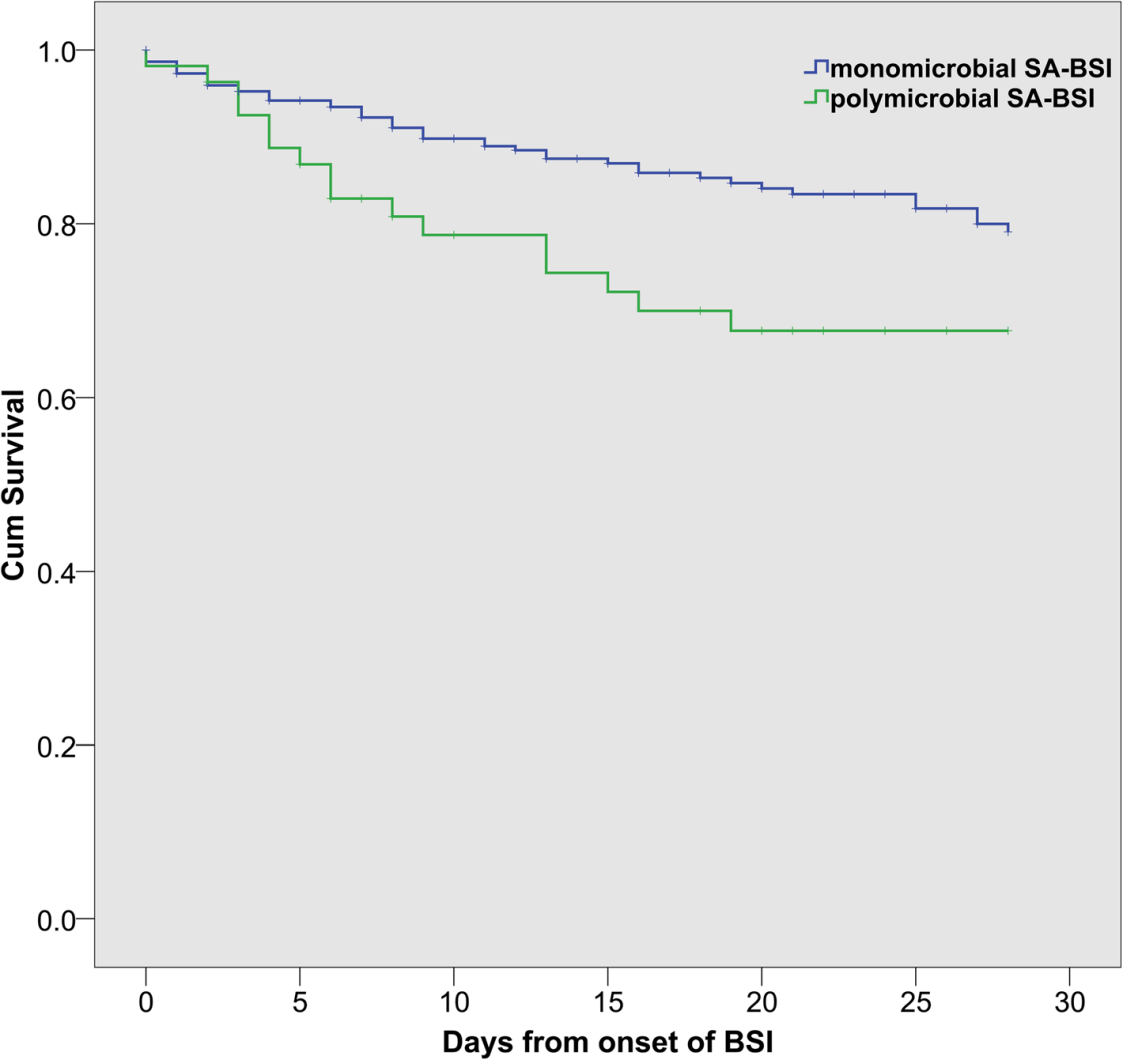
Kaplan-Meier estimates of survival in patients with polymicrobial *Staphylococcus aureus* bloodstream infections and monomicrobial *Staphylococcus aureus* bloodstream infections. Abbreviations: SA-BSI, *Staphylococcus aureus* bloodstream infections

In comparison with monomicrobial SA-BSI, patients with polymicrobial SA-BSI had a longer length of hospital stay [median days, 28(15–49) vs. 50(21.25–85.75), *p* < 0.01], and ICU stay [median days, 0(0–12) vs. 23(6.25–49.25), *p* < 0.01] (Table 5). Sepsis occurred in 72.2% of polymicrobial SA-BSI and in 84.4% of monomicrobial SA-BSI (*p* = 0.03), whereas the occurrence rate of septic shock in patients with polymicrobial SA-BSI was almost two-fold higher than that with monomicrobial SA-BSI (16.7% vs. 8.8%, *p* = 0.08). The overall in-hospital crude mortality rate was 20.3%, which was significantly higher in patients with polymicrobial SA-BSI than that in patients with monomicrobial SA-BSI (31.5% vs. 18.3%, *p* = 0.03). Like the 28-day mortality (29.6% vs. 15.3%, *p* = 0.01), the 7-day and 14-day mortalities in patients with polymicrobial SA-BSI were also significantly higher than those with monomicrobial SA-BSI (16.7% vs. 7.5%, *p* = 0.03; 24.1% vs. 11.2%, *p* = 0.01, respectively) (Table 5), which were consistent with the results from the survival curves of patients in both groups (Fig. 3).

**Table 5:** Comparison of outcome between monomicrobial SA-BSI and polymicrobial SA-BSI

Prognostic indicators	Total (*n* = 349)	Mono-SA-BSI (*n* = 295)	Poly-SA-BSI (*n* = 54)	*p*-value
Total Hospitalization days (M) (IQR)	30 (17,54.5)	28 (15,49)	50 (21.25,85.75)	*0.00*
Total ICU residence days (M) (IQR)	1 (0,18)	0 (0,12)	23 (6.25,49.25)	*0.00*
Cause sepsis	288 (82.5%)	249 (84.4%)	39 (72.2%)	*0.03*
Cause Septic shock (n,%)	35 (10%)	26 (8.8%)	9 (16.7%)	0.08
7 day total mortality rate (n,%)	31 (8.9%)	22 (7.5%)	9 (16.7%)	*0.03*
14 day total mortality rate (n,%)	46 (14.2%)	33 (11.2%)	13 (24.1%)	*0.01*
28 day total mortality rate (n,%)	61 (17.5%)	45 (15.3%)	16 (29.6%)	*0.01*
In-hospital mortality (n,%)	71 (20.3%)	54 (18.3%)	y (31.5%)	*0.03*

*Abbreviations*: M Median, *IQR* Interquartile range, *ICU* Intensive care unit, *SA-BSI Staphylococcus aureus* bloodstream infections

## Discussion

In the current study, several important results were found. First, polymicrobial SA-BSI are not rare among *Staphylococcus aureus* bacteremia. Second, some risk factors were found to be associated with polymicrobial SA-BSI as shown in Table 1. Moreover, burn injury, prior blood transfusion, mechanical ventilation, pneumonia as a primary site of infection, and length of prior hospital stay were independent risk factors for polymicrobial SA-BSI (Table 3). Third, *A. baumannii* was the most common co-pathogen in polymicrobial SA-BSI, followed by *Enterococcus* spp.. Last, patients with polymicrobial SA-BSI might have worse outcomes including higher occurrence of septic shock, prolonged ICU stay, and hospital stay as well as higher mortality in comparison with monomicrobial SA-BSI.

A high proportion of 15.5% was polymicrobial SA-BSI among SA-BSI in the current study, which was consistent with previous studies that polymicrobial bacteremia accounts for 5–20% of bloodstream infection [7, 18–20]. In Park’s report [10], the polymicrobial SA-BSI was accounted for 9.6% (44/456) of all episodes of BSI in a tertiary referral center of Korea. A 6.1% (93/1537) frequency of polymicrobial SA-BSI was reported in a 772-bed teaching hospital in Michigan in Khatib’s study [11]. In our recently previous study [21], we also found that 34.8% cases (157/451) with enterococcal bloodstream infections were mixed with other pathogens like CNS, *A. baumannii* and *Klebsiella pneumoniae*. These studies suggest that the proportion of polymicrobial bloodstream infections is not rare, which deserves the attention of clinicians.

We found that clinical and demographic were different between the monomicrobial and polymicrobial groups. This suggests that many factors were associated with polymicrobial SA-BSI (Table 1). One of them appears to be patients with traumatic or burn injuries leading to neurologic deficits, prolonged ventilation and ICU stays, and frequent antibiotics (as evidenced by higher resistance rates, Table 4). Many independent risk factors for polymicrobial SA-BSI were observed in our current study including burn injury, prior blood transfusion, mechanical ventilation, pneumonia as a primary site of infection, and length of prior hospital stay. A previous study has shown that more than 12% of burn patients suffered from polymicrobial BSI [22]. Our previous study focusing on enterococcal bloodstream infection also confirmed that burn was an independent risk factor for mixed-enterococcal bloodstream infections [21]. As described in previous studies [23–25], burn patients were at a high risk of BSI as they encountered alterations in cellular and humoral immune responses, extensive skin barrier disruption, high possibility of gastrointestinal bacterial translocation, prolonged hospitalization, and invasive diagnostic/therapeutic procedures. Therefore, as the most common colonizing pathogens of the skin, *S. aureus* is more likely to invade the blood through the skin of burn patients and caused bloodstream infections together with other pathogens.

Blood transfusion was an independent risk factor of polymicrobial SA-BSI in our research, which was consistent with previous study, showing that transfusion of red blood cells and platelets was associated with the onset of secondary bacterial infection in sepsis patients [26]. This is partly explained that transfusion can cause persistent immune dysfunction in mouse model of sepsis [27] and the fact that immunosuppressive patients are more likely to have polymicrobial BSI [20, 28]. In addition, patients with polymicrobial SA-BSI were more severe evidenced by high APACHE II score and SOFA score (Table 1), which suggested that these patients were more like in the immunosuppressive state.

Our study also showed mechanical ventilation and pneumonia as a primary site of infection were independent factors of polymicrobial SA-BSI. As shown in the current study, pneumonia was the most common source of SA-BSI and was significantly more frequent among patients with polymicrobial than monomicrobial SA-BSI, which was consistent with Sancho’s study showing that lower respiratory tract was the main source of BSI [9]. In contrast, a previous study [10] has shown that intra-abdominal infections were the most common source of polymicrobial infection, but a selected bias might be existed as a high proportion (7%) of biliary tract disease was observed in their institution. Compared with monomicrobial SA-BSI, our study showed that polymicrobial SA-BSI had a higher APACHE II score, a higher SOFA score, a higher Pitt Bacteremia score, and more frequent admission in ICU (Table 1). These results suggested that polymicrobial SA-BSI was associated with more severity conditions. Critical patients with pneumonia might tend to develop respiratory failure and require mechanical ventilation [8], and mechanical ventilation can increase the incidence of ventilators-associated complications, such as ventilators associated pneumonia (VAP) [29, 30], leading to recurrent lung infections and the increased possibility of polymicrobial bloodstream infections. Like in our and Sancho’s study [9, 21], an independent association between the days of prior hospital stay and polymicrobial infection was also observed in the current study. It can be inferred that such patients would be more predisposed to health care exposure and risks for contracting polymicrobial infection. Thus, it might be important to reduce unnecessary interventions and shorten the length of stay for patients.

The most common co-pathogen in polymicrobial SA-BSI was *A. baumannii* (27.9%) in the current study. In fact, the high proportion of *A. baumannii* as co-pathogens in polymicrobial SA-BSI is also indirectly reflected by the evidence that pneumonia as a primary site of infection and mechanical ventilation were independent risk factors for polymicrobial SA-BSI in our current study, as *A. baumannii* was frequently associated with pneumonia infection especially for VAP [31, 32]. By the way, *A. baumannii* as the most common pathogen in post-neurosurgical intracranial infections accounted for 38.8 percentage in our previous study [33]. These results mean *A. baumannii* is a real threat for hospital-acquired infection. Patients with polymicrobial SA-BSI might have worse outcomes than those with monomicrobial SA-BSI, including prolonged lengths of ICU stay and hospital stay, the 28-day mortality, which were consistent with previous reports [9, 10]. Although early appropriate antimicrobial therapy has been shown to reduce mortality among bacteremia patients [34], there was no difference in delayed antibiotic therapy between the two groups in our study. The worse outcomes of polymicrobial SA-BSI in our study were possibly associated with the following factors: (1) the proportion of septic shock in patients with polymicrobial SA-BSI was two-fold higher than that with monomicrobial SA-BSI (16.7% vs. 8.8%), though there was no statistical difference. (2) Interestingly, MRSA was significantly more frequent in patients with polymicrobial than monomicrobial SA-BSI (81.5% vs. 65.1%, *p* = 0.018). As shown in a previous meta-analysis, methicillin resistance is associated with increased mortality in patients with *S. aureus* bacteremia [35]. (3) A high proportion of secondary bloodstream infections was observed in polymicrobial SA-BSI than that in monomicrobial SA-BSI (90.7% vs. 70.2%). Previous study has shown that the risk of mortality associated with primary bacteremia like the catheter-related bloodstream infection appears much lower than that of secondary bloodstream infections [36].

However, there were some limitations in the present study. First, it was a retrospective study, and as a result, the patient characteristics, co-morbidities, and some other information were obtained based on the review of patient records rather than an interview or clinical examination at the time of infection, which might lead to some important information or variable such as Glasgow coma scale score could not be obtained accurately. Second, the current study was performed from a single center and the number of patients was relatively small, though it has reviewed the record of SA-BSI over a 6-year period in our hospital. In addition, our institution is well-known in the field of trauma treatment nationwide, there was a considerable number of patients with trauma and burn in the study, which might lead to selection bias. Thus, the results from the current study might not be suitable for other hospitals. Third, it is possible that some important confounding variables for polymicrobial SA-BSI were not included and analyzed, as its intrinsic shortcoming from retrospective study. Thus, a multi-centric study with a large sample size is necessary to further investigate the risk factors of polymicrobial SA-BSI for better prevention.

## Conclusions

Polymicrobial SA-BSI is not a few events among *Staphylococcus aureus* bacteremia, and *Acinetobacter baumannii* is the predominant co-existed species. Burn injury, blood transfusion, mechanical ventilation, the length of prior hospital stay, and pneumonia as a primary site of infection are independent risk factors for polymicrobial SA-BSI. In addition, patients with polymicrobial SA-BSI might have worse outcomes compared with monomicrobial SA-BSI, which might be attracted more attention by physicians in the future.

## Data Availability

All data generated or analyzed during this study are included in this manuscript.

## Abbreviations

SA-BSI: *Staphylococcus aureus* bloodstream infections
CCI: Charlson Comorbidity Index
OR: Odds ratio
CI: Confidence interval
ICU: Intensive care unit
BSI: Bloodstream infections
CNS: Coagulase-negative *Staphylococcus*
GPT: Glutamic-pyruvic transaminase
GOT: Glutamic-oxaloacetic transaminase
APACHE: Acute physiology and chronic health evaluation
SOFA: Sequential organ failure assessment
CDC: Centers for Disease Control and Prevention
IQR: Interquartile range
MRSA: Methicillin-resistant *Staphylococcus aureus*
CLSI: Clinical and Laboratory Standards Institute
*A. baumannii*: *Acinetobacter baumannii*
*S. aureus*: *Staphylococcus aureus*
