# Correction: Clinical characteristics and risk factors of polymicrobial *Staphylococcus* *aureus* bloodstream infections

**DOI:** 10.1186/s13756-022-01193-w

**Published:** 2022-12-08

**Authors:** Cheng Zheng, Shufang Zhang, Qingqing Chen, Li Zhong, Tiancha Huang, Xijiang Zhang, Kai Zhang, Hongwei Zhou, Jiachang Cai, Linlin Du, Changming Wang, Wei Cui, Gensheng Zhang

**Affiliations:** 1 Department of Critical Care Medicine, Second Affiliated Hospital, Zhejiang University School of Medicine, Hangzhou, 310009 China; 2 Department of Critical Care Medicine, Taizhou Municipal Hospital, Taizhou, 318000 Zhejiang China; 3 Department of Cardiology, Second Affiliated Hospital, Zhejiang University School of Medicine, Hangzhou, 310009 China; 4 Department of Critical Care Medicine, Taizhou Enze Medical Center (Group) Enze Hospital, Taizhou, 318050 Zhejiang China; 5 Department of Critical Care Medicine, Huzhou First People’s Hospital, Huzhou, 313000 Zhejiang China; 6 Clinical Microbiology Laboratory, Second Affiliated Hospital, Zhejiang University School of Medicine, Hangzhou, 310009 China

**Correction: Antimicrobial Resistance & Infection Control (2020) 9:76** https://doi.org/10.1186/s13756-020-00741-6

The original article [1] contains an error in Table 5 in column ‘Poly-SA-BSI (n = 54)’, row ‘In-hospital mortality (n,%)’.

The current, incorrect value is ‘y (31.5%)’ whereas the correct value is instead ’17 (31.5%)’.

## References

[1] Zheng (2020). Clinical characteristics and risk factors of polymicrobial *Staphylococcus*
*aureus* bloodstream infections. Antimicrob Resist Infect Control.

